# Predicting survival in oral squamous cell carcinoma via integrated analysis of tumor budding and tertiary lymphoid structures

**DOI:** 10.3389/fonc.2026.1774998

**Published:** 2026-06-03

**Authors:** Zhuqin Xiang, Yaqi Huang, Bokai Yun, Dongpeng Li, Fengshuo Liu, Junliang Liu, Nan Xie, Zehang Zhuang, Jinsong Hou, Cheng Wang

**Affiliations:** 1 Hospital of Stomatology, Sun Yat-sen University, Guangzhou, China; 2 Guangdong Provincial Key Laboratory of Stomatology, Guangzhou, China; 3 Guanghua School of Stomatology, Sun Yat-sen University, Guangzhou, China

**Keywords:** oral squamous cell carcinoma, retrospective cohort analysis, risk stratification, tertiary lymphoid structures, tumor budding

## Abstract

**Background:**

Oral squamous cell carcinoma (OSCC) poses a significant health burden. Accumulating evidence indicates that tumor budding (TB) and tertiary lymphoid structures (TLS) are both associated with the survival prognosis of OSCC patients; however, the prognostic value of integrated TB and TLS has not been investigated. The objective of this study was to validate the prognostic value of TB and TLS in OSCC, establish a TB-TLS interaction model to assess the “invasion-immune” status in OSCC tumor microenvironment.

**Patients and methods:**

A total of 510 patients diagnosed with OSCC were included in this retrospective study. TB and TLS were assessed via hematoxylin and eosin staining, and a TB/TLS index was established. Survival analysis and Cox regression were performed using R software, and a nomogram prediction model incorporating the TB/TLS index with clinicopathological factors was constructed and validated.

**Results:**

High-grade TB correlated with poor survival, while high-maturation TLS indicated favorable outcomes. The TB/TLS index exhibited superior prognostic discriminatory ability versus single biomarkers. Furthermore, a nomogram incorporating the TB/TLS index was developed and validated, demonstrating reliable predictive performance and good calibration.

**Conclusions:**

This retrospective study validated the prognostic value of TB and TLS in OSCC. The TB/TLS index showed better discriminative ability. External validation demonstrated that the nomogram prediction model achieved moderate predictive performance despite substantial baseline differences between cohorts.

## Introduction

Head and neck malignancies rank among the top ten most common cancers worldwide, involving anatomical sites such as the oral cavity, pharynx, and larynx. The predominant pathological type is squamous cell carcinoma, with oral squamous cell carcinoma (OSCC) being a major subtype of head and neck malignancies (1). Global cancer statistics in 2022 revealed that OSCC accounts for 389,485 new cases and 188,230 related deaths annually, with its disease burden being particularly prominent in developing countries (1). Currently, surgical resection remains the mainstay of clinical treatment for OSCC, supplemented by comprehensive therapeutic modalities such as radiotherapy and chemotherapy. Although significant advances have been achieved in immunotherapy and targeted therapy in recent years, the 5-year survival rate of OSCC patients remains suboptimal (2). This indicates that further research is warranted regarding the selection of diagnosis and treatment strategies and the prediction of prognosis for OSCC patients.

The tumor microenvironment (TME) is a highly structured system consisting of the extracellular matrix (ECM), tumor cells, immune cells, stromal cells, and signal molecules secreted by cells. It plays a crucial regulatory role in the occurrence, development, and metastasis of cancer (3). Tumor budding (TB), a key component of the TME, is defined as isolated single cancer cells or cell clusters composed of fewer than 5 cancer cells located at the tumor invasive front (4). Its pathological characteristics include the loss of intercellular junctions and dedifferentiation. It is closely associated with tumor invasion, metastasis, and poor prognosis in patients with various solid tumors, including OSCC (5–7). Biologically, TB cells exhibit partial epithelial-mesenchymal transition (EMT) and stem cell-like characteristics (8), and play a pivotal role in the cancer metastasis cascade, suggesting that TB represents the “offensive” mode of tumors.

In recent years, several studies have successively reported the aggregation of B lymphocyte follicles and the infiltration of T lymphocytes outside the follicles in the TME, forming structures similar to germinal centers (9, 10). Such ectopic lymphoid organs that develop from non-lymphoid tissues in a chronic inflammatory environment are termed tertiary lymphoid structures (TLS). Mature TLS are similar to secondary lymphoid organs in terms of structure and function (11), and have been confirmed to directly or indirectly participate in and regulate anti-tumor immune responses in various solid tumors, including OSCC, thereby playing a positive role in inhibiting tumor progression (12–14). Therefore, in contrast to the pro-tumor properties of TB, TLS in the TME represent a “defensive” response of the body in patients.

Studies have indicated that constructing an “attacker-defender” model based on the analysis of pro-tumor and anti-tumor factors in the TME to generate novel prognostic predictors can improve the prediction of lymph node metastasis and survival in patients (15). By integrating the analysis of TB and TLS to form an “attacker-defender” counteraction model, we can evaluate the dynamic balance between tumors and the immune system. In this study, based on the TB-TLS counteraction model and relevant clinicopathologic features, we developed a nomogram-based survival prediction model to visualize the risks of multiple prognostic factors.

## Materials and methods

### Study cohort

This study received approval from the ethical committee of Hospital of Stomatology, Guanghua School of Stomatology, Sun Yat-Sen University (KQEC-2025-100-01), and the privacy of all patients was fully protected. The samples analyzed in this study included a retrospective training cohort (Cohort A) and a retrospective validation cohort (Cohort B), derived from the Hospital of Stomatology, Sun Yat-sen University and The Cancer Genome Atlas (TCGA) public database, respectively. All patients were restaged according to the 8th edition American Joint Committee on Cancer (AJCC) tumor, node, metastasis (TNM) staging system.

As the primary study cohort, Cohort A included 290 OSCC patients who underwent surgical resection at the Hospital of Stomatology, Sun Yat-sen University, between January 2010 and December 2017. The inclusion and exclusion criteria were as follows. Inclusion criteria: (1) Histopathological confirmation of OSCC; (2) Available qualified pathological sections; (3) Complete medical records and feasibility of follow-up; (4) Availability of qualified pathological sections. Exclusion criteria: (1) Prior history of head and neck cancer or previous receipt of any treatment; (2) Comorbidity with other malignant tumors.

Cohort B were obtained from the Genomic Data Commons (GDC) platform of the National Cancer Institute (https://portal.gdc.cancer.gov/) and the TCGA public database. We downloaded the clinicopathological information, follow-up data, and hematoxylin and eosin (H&E)-stained diagnostic pathological sections of OSCC patients from this platform, and ultimately selected 220 eligible OSCC patients. The inclusion and exclusion criteria were as follows. Inclusion criteria: (1) Histopathological confirmation of OSCC; (2) Available qualified pathological sections; (3) Complete medical records and feasibility of follow-up; (4) Availability of H&E-stained diagnostic pathological sections. Exclusion criteria: (1) Prior history of head and neck cancer or previous receipt of any treatment; (2) Comorbidity with other malignant tumors.

### Follow-up and clinical outcome

Clinical data of Cohort A, including survival and recurrence status, were obtained from patients’ follow-up medical records and telephone follow-up. Clinical data of Cohort B were retrieved from the TCGA database. The primary endpoint of this study was overall survival (OS), defined as the time from surgical resection to death from any cause, with censoring at the last follow-up. The secondary endpoint was disease-free survival (DFS), defined as the time from surgical resection to recurrence at any site or death from any cause.

### Histopathological assessment

All H&E-stained pathological sections were scanned and processed using the Leica Aperio ScanScope AT2 slide scanner, along with eSlide Manager and ImageScope (Leica Biosystems) software. Tissue integrity, staining quality, and the presence of the tumor invasive front were checked.

TB assessment was independently performed by two authors, following the recommendation of the 2016 International Tumor Budding Consensus Conference (ITBCC) (16). Briefly, the invasive front of all slides was inspected under 100× magnification to identify the hot spot with the highest TB density. Subsequently, an area of 0.785 mm² in the hot spot was selected, and TB was counted under 200× magnification. Based on the TB count, cases were graded as Bd1 (0–4 buds), Bd2 (5–9 buds), and Bd3 (≥10 buds). Referring to previous relevant studies (17), Bd2 and Bd3 were defined as high-grade TB, while Bd1 was defined as low-grade TB in this study. For cases in which determination of TB grade was challenging, the two authors reviewed the sections together and reached a consensus.

The highest maturation level of TLS was independently assessed by two authors through observation of H&E-stained pathological sections. Based on their maturation levels, TLS were classified into three stages: early tertiary lymphoid structures (E-TLS), primary follicle-like tertiary lymphoid structures (PFL-TLS), and secondary follicle-like tertiary lymphoid structures (SFL-TLS). Specifically, E-TLS are dense lymphocyte aggregates lacking follicular dendritic cells (FDC); PFL-TLS have an FDC network but no germinal centers (GC); SFL-TLS possess both an FDC network and GC (18). We acknowledge that the distinction between E-TLS and PFL-TLS—specifically the presence of an FDC network—cannot be reliably determined on H&E alone. Therefore, in this study, the classification of PFL-TLS was based on the presence of a well-organized lymphoid follicle without a germinal center, recognizing that this may overestimate E-TLS and underestimate true PFL-TLS. According to the highest TLS maturation level, patients were stratified into a low-maturation group (absence of TLS or presence of E-TLS) and a high-maturation group (presence of PFL-TLS or SFL-TLS). In cases where discrepancies arose regarding TLS maturation levels, the two authors reviewed the sections together and reached a consensus.

The pathological differentiation was classified according to the classification criteria of World Health Organization. The depth of invasion (DOI) was defined as the vertical distance from the basement membrane of the normal epithelial mucosa to the deepest point of tumor invasion.

### Statistical analyses

Statistical analyses in this study were performed using R software (Version 4.5.2), with the following R packages employed: “survival” for Cox regression analysis, “survminer” for generating survival curves, “timeROC” for calculating receiver operating characteristic (ROC) curves at specific time points, “optimx” for adjusting parameters to identify optimal combined weights, and “ggplot2” for data visualization. A two-tailed p-value < 0.05 was considered statistically significant for all analyses.

For patient characteristics in this study, continuous variables were expressed as median (range), and categorical variables as frequencies (percentages). Comparisons between groups were performed using the chi-square test or Fisher’s exact test, as appropriate.

The Kaplan-Meier method was used to estimate OS and DFS after primary surgical treatment, with the log-rank test conducted to calculate chi-square values and survival curves plotted. Cox proportional hazards regression was applied for univariate and multivariate analyses to identify independent prognostic factors. The proportional hazards assumption was verified using Schoenfeld residuals (p > 0.05 for all covariates). Hazard ratios (HRs) with 95% confidence intervals (CIs) were reported.

To avoid methodological circularity, the entire Cohort A was randomly split into a model training set (70%) and an independent test set (30%) using a random seed for reproducibility. All index construction, parameter optimization, and cutoff determination were performed exclusively in the training set; the test set remained completely untouched until final validation. To construct the TB/TLS index, six candidate forms were evaluated in the training set, including simple ratio (TB/TLS), log-transformed ratio (log(TB/TLS)), inverse ratio (TLS/TB), difference (TB−TLS), TB alone, and TLS alone. The form with the highest Harrell’s C-index and log-rank χ² was selected as the final index.

The optimal cutoff value was determined using “10-fold cross-validation” in the training set to ensure stability. This fixed cutoff was then applied without modification to the independent test set and the external validation cohort (Cohort B).

### Construction and validation of nomogram prediction model

Based on the results of multivariate Cox proportional hazards regression model analysis, independent risk factors and protective factors were screened, and the “rms” package was applied to construct a Nomogram prediction model. In the prediction model, clinically relevant 1-year, 3-year, and 5-year OS were selected as the prognostic time points, and the contribution of each predictive variable as well as the survival probability were presented. The likelihood ratio test (LRT) was used to determine whether the prediction model incorporating the TB/TLS index was significantly superior to the baseline model. The Akaike information criterion (AIC)/Bayesian information criterion (BIC) was used to test the goodness of fit and parsimony of the model via difference analysis; the smaller the AIC/BIC value, the better the model performance.

Validation of the Nomogram model included two parts: internal validation and external validation. For internal validation, the Bootstrap method (1000 times of resampling with replacement) was used to plot calibration curves and calculate the concordance index (C-index). The closer the calibration curve was to the ideal prediction line, the higher the consistency between the predicted results and actual observations. For external validation, independent cohort data were used to evaluate the discrimination and calibration of the model; the “timeROC” package was applied to plot time-dependent ROC curves, calculate the time-dependent area under the ROC curve (AUC), and generate calibration curves to assess the generalization ability of the model.

## Result

### Clinicopathological characteristics of the OSCC cohort

A total of 510 patients were enrolled in this study, derived from two independent databases, of whom 290 were recruited from the Hospital of Stomatology, Sun Yat-sen University (Cohort A) and 220 from TCGA public dataset (Cohort B). The characteristics of the study participants are shown in **Table 1**.

**Table 1:** Clinicopathologic characteristics of cohorts.

Clinicopathologic features	Cohort A, N = 290	Cohort B, N = 220	*p*
	Frequency, No. (%)	Frequency, No. (%)	
Age, years			<0.001*
≥ 60	97 (33.5)	124 (56.4)	
< 60	193 (66.5)	96 (43.6)	
Gender			0.291
Female	108 (37.2)	72 (32.7)	
Male	182 (62.8)	148 (67.3)	
Tumor site			<0.001*
Tongue	178 (61.4)	87 (39.5)	
Others	112 (38.6)	133 (60.5)	
Pathological T stage			<0.001*
pT1–2 stage	243 (83.8)	84 (38.2)	
pT3–4 stage	47 (16.2)	136 (61.8)	
DOI			<0.001*
≤ 5mm	155 (53.5)	26 (17.4)	
> 5mm	135 (46.5)	123 (82.6)	
TNM stage			<0.001*
I/II stage	219 (75.5)	62 (28.2)	
III/IV stage	71 (24.5)	158 (71.8)	
Pathologic differentiation			<0.001*
Well	149 (51.4)	36 (16.4)	
Moderately/Poorly	141 (48.6)	184 (83.6)	
Lymph node metastasis			<0.001*
Yes	44 (15.2)	97 (44.1)	
No	246 (84.8)	123 (55.9)	
Relapse			0.344
Yes	76 (26.2)	66 (30.0)	
No	214 (73.8)	154 (70.0)	
TB grade			0.081
low grade	116 (40.0)	105 (47.7)	
high grade	174 (60.0)	115 (52.3)	
TLS maturity			0.023*
None/E-TLS	211 (72.8)	179 (81.4)	
PFL-TLS/SFL-TLS	79 (27.2)	41 (18.6)	

Depth of invasion status can be evalued for only 164 patients in validation cohort, *represents p<0.05.

DOI, depth of invasion; TNM, tumor-lymph node-metastasis; TB, tumor budding; TLS, tertiary lymphoid structures.

In Cohort A, 182 patients were male (62.8%) and 108 were female (37.2%). The age of patients at diagnosis ranged from 19 to 87 years, with a median of 54 years. The tongue was the most common primary tumor site, accounting for 178 cases (61.4%). According to the 8th edition AJCC TNM staging system, 243 patients (83.8%) were diagnosed as pT1-2, and 47 patients (16.2%) as pT3-4. Regarding the DOI, 155 cases (53.5%) had a DOI ≤ 5 mm, and 135 cases (46.5%) had a DOI > 5 mm, with a median DOI of 4.70 mm (range: 0.30 – 21.03 mm). There were 149 cases (51.4%) of well differentiated, 141 cases (48.6%) of moderately/poorly differentiated. According to the follow-up records, the follow-up duration ranged from 5 to 123 months, with a median of 72 months. At the last follow-up, 76 patients (24.2%) had postoperative recurrence, and 77 patients (26.6%) died. Among the recurrent patients, 53 cases (18.3%) had local recurrence, 31 cases (10.7%) had regional lymph node metastasis, and 8 cases (2.8%) had distant metastasis. The median times to local recurrence, regional lymph node metastasis, and distant metastasis were 17 months, 8 months, and 16 months, respectively.

In Cohort B, 148 patients were male (67.3%) and 72 were female (32.7%). The age at diagnosis ranged from 24 to 88 years, with a median of 61 years. Eighty-seven cases (39.5%) had tumors located in the tongue, which was the most common site among those with clearly documented tumor locations. According to the 8th edition AJCC TNM staging system, 84 patients (38.2%) were classified as pT1-2, and 136 patients (61.8%) as pT3-4. Only 149 cases in this cohort had evaluable DOI, among them, 26 cases (17.4%) had a DOI ≤ 5 mm, and 123 cases (82.6%) had a DOI > 5 mm, with a median DOI of 7.93 mm (range: 1.63 – 19.35 mm). There were 36 cases (16.4%) of well differentiated, 184 cases (83.6%) of moderately/poorly differentiated. According to the follow-up records, the follow-up duration ranged from 4 to 183 months, with a median of 23.5 months. At the last follow-up, 66 patients (30.0%) had postoperative recurrence, and 101 patients (45.9%) died. Among the recurrent patients, 34 cases (15.5%) had local recurrence, 19 cases (8.6%) had regional lymph node metastasis, and 15 cases (6.8%) had distant metastasis. The median times to local recurrence, regional lymph node metastasis, and distant metastasis were 8 months, 6 months, and 9 months, respectively.

**Figure 1:**
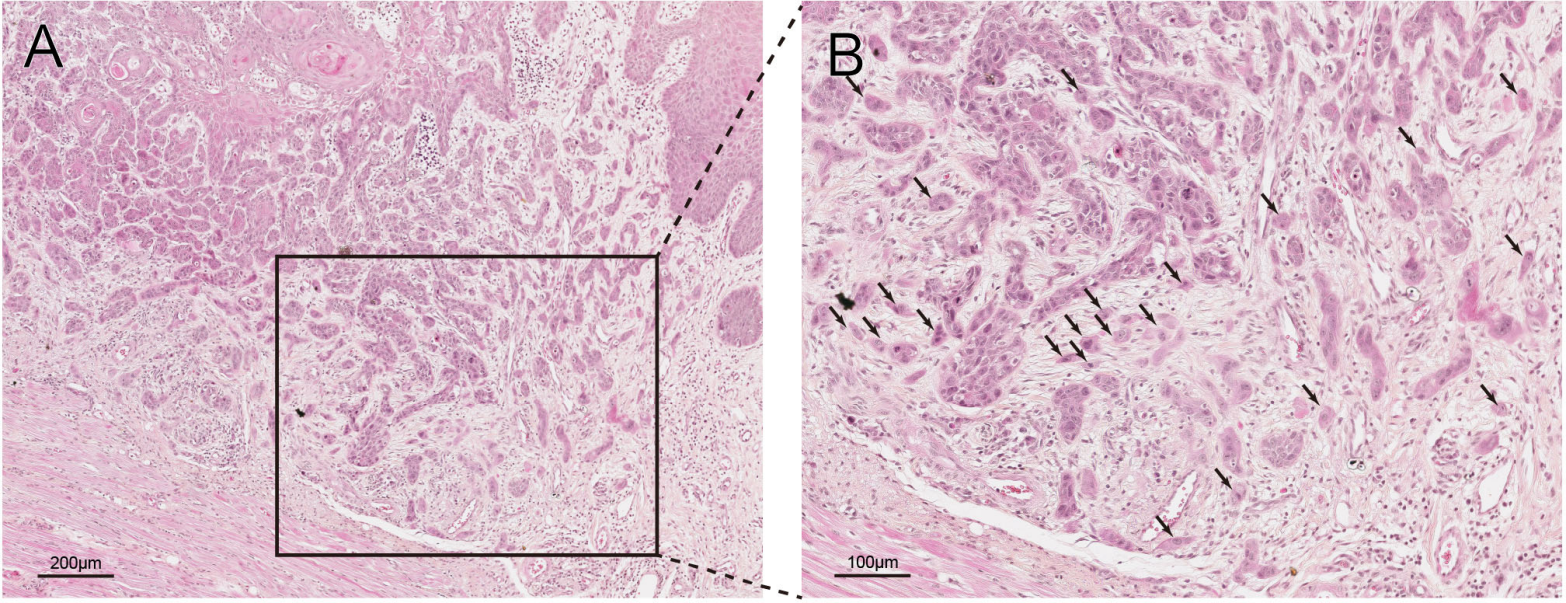
Tumor budding at the invasive tumor front in oral squamous cell carcinoma. **(A)** H&E-stained section at ×100 magnification, scale bar: 200 μm; **(B)** H&E-stained section at ×200 magnification, arrows indicate tumor budding, scale bar: 100 μm.

Tumor buds can be readily identified based on standard H&E staining as shown in **Figure 1**. According to the ITBCC classification criteria, 116 patients (40.0%) in Cohort A were classified as Bd1, 110 patients (37.9%) as Bd2, and 64 patients (22.1%) as Bd3. In Cohort B, 105 patients (47.7%) were Bd1, 67 patients (30.5%) were Bd2, and 48 patients (21.8%) were Bd3.

**Figure 2:**
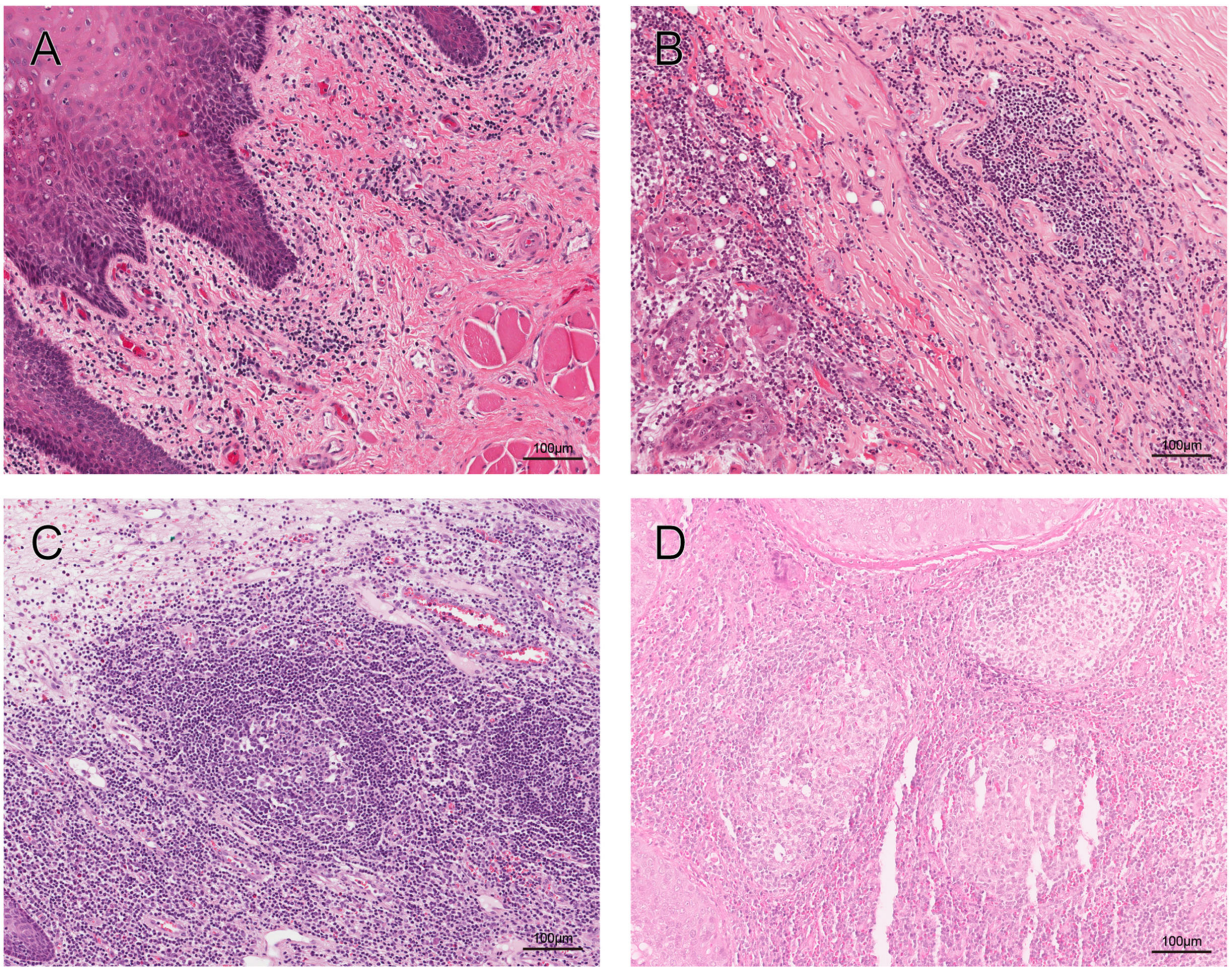
Tertiary lymphoid structures in oral squamous cell carcinoma. **(A)** Absence of tertiary lymphoid structures; **(B)** Early-stage tertiary lymphoid structures, formed by dense lymphocyte aggregates; **(C)** Primary follicle-like tertiary lymphoid structures, composed of B-cell clusters with a follicular dendritic cell network; **(D)** Secondary follicle-like tertiary lymphoid structures, with visible germinal centers in the center of lymphoid follicles. All images are hematoxylin and eosin-stained sections at ×200 magnification, scale bar: 100 μm.

As shown in **Figure 2**, TLS could be identified and their maturation levels can be evaluated using standard H&E-stained pathological sections. In Cohort A, 63 patients (21.7%) had no TLS, 148 patients (51.0%) had E-TLS, 58 patients (20.0%) had PFL-TLS, and 21 patients (7.3%) had SFL-TLS. In Cohort B, 60 patients (27.3%) had no TLS, 119 patients (54.1%) had E-TLS, 24 patients (10.9%) had PFL-TLS, and 17 patients (7.7%) had SFL-TLS. Referring to the classification methods of previous studies, patients were stratified into the low-maturation group (no TLS or E-TLS) and high-maturation group (PFL-TLS or SFL-TLS) based on TLS maturation levels.

Interobserver reproducibility for TB grading and TLS maturation scoring was evaluated in Cohort A and Cohort B, respectively. For TB grade, the kappa value was 0.78 (95% CI: 0.72–0.84) in Cohort A and 0.79 (95% CI: 0.72–0.86) in Cohort B, showing substantial to excellent agreement. For TLS maturation grading, the kappa value was 0.69 (95% CI: 0.62–0.76) in Cohort A and 0.67 (95% CI: 0.60–0.76) in Cohort B, demonstrating moderate to substantial agreement. These results confirm the reliability and consistency of the histopathological scoring system.

Chi-square tests were performed to compare the clinicopathologic characteristics between the two cohorts. Significant differences were observed in the clinicopathologic features between the two cohorts, which reflect inherent heterogeneity between a single-institution retrospective cohort and a public database cohort. Compared with Cohort A, Cohort B had patients with older age, higher pathological T and TNM stages, greater DOI, a higher proportion of moderately/poorly differentiated cases, more frequent lymph node metastasis, and a lower proportion of cases with PFL-TLS or SFL-TLS. No significant differences were found in gender distribution, postoperative recurrence status, or TB grade between the two cohorts.

### Correlation of TB and TLS with clinical outcomes in OSCC

**Figure 3:**
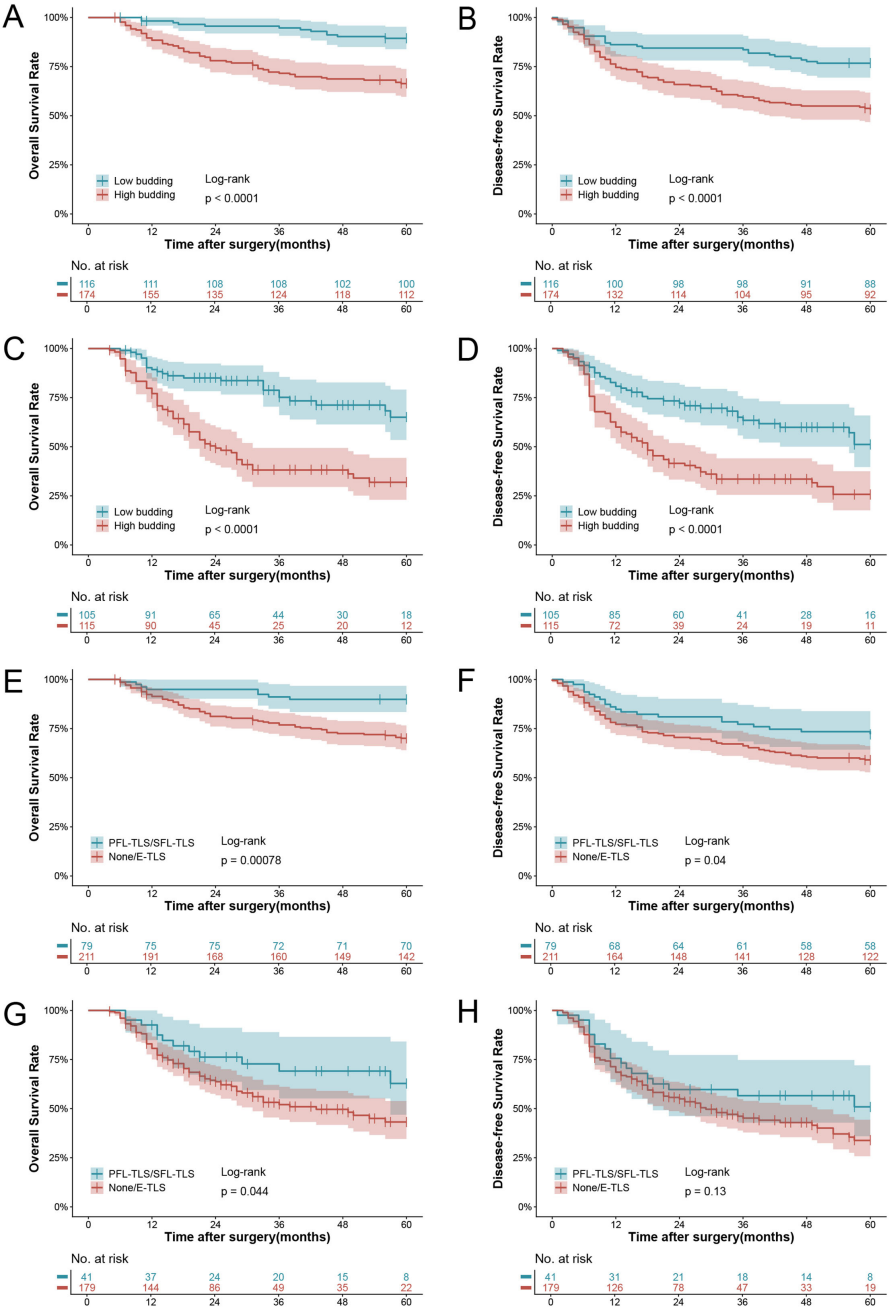
Kaplan-Meier curves for OS and DFS of OSCC patients. **(A, B)** OS and DFS curves stratified by TB grade in OSCC patients from cohort A; **(C, D)** OS and DFS curves stratified by TB grade in OSCC patients from cohort B; **(E, F)** OS and DFS curves stratified by TLS maturation status in OSCC patients from cohort A; **(G, H)** OS and DFS curves stratified by TLS maturation status in OSCC patients from cohort B The colored areas in the figures represent the 95% confidence intervals. OS, overall survival; DFS, disease-free survival; OSCC, oral squamous cell carcinoma; TB, tumor budding; TLS, tertiary lymphoid structures; None indicates absence of tertiary lymphoid structures; E-TLS, early tertiary lymphoid structures; PFL-TLS, primary follicle-like tertiary lymphoid structures; SFL-TLS, secondary follicle-like tertiary lymphoid structures.

Survival analyses were performed based on TB grade and TLS maturation level, with survival curves plotted and log-rank tests conducted. In both Cohort A and Cohort B, the 5-year OS rates in the high-grade TB group were 66.4% and 31.9%, respectively, which were significantly lower than those in the low-grade TB group (89.4% and 65.0%, respectively; p < 0.0001 for both; **Figures 3A, C**). Similarly, the 5-year DFS rates in the high-grade TB group were 53.2% and 25.7% in Cohort A and Cohort B, respectively, which were significantly lower than those in the low-grade TB group (76.7% and 51.1%, respectively; p < 0.0001 for both; **Figures 3B, D**). Regarding TLS maturation, the 5-year OS rates in the PFL-TLS/SFL-TLS group were 89.9% and 62.9% in Cohort A and Cohort B, respectively, which were significantly higher than those in the None/E-TLS group (70.0% and 43.2%, respectively; p = 0.00078 and p = 0.044; **Figure 3E**, **Figure 3G**). In Cohort A, the DFS rate in the PFL-TLS/SFL-TLS group was 72.2%, which was higher than that in the None/E-TLS group (59.1%, p = 0.04; **Figure 3F**). However, no significant difference in DFS was observed between those two groups in Cohort B (p = 0.13; **Figure 3H**).

As shown in **Table 2**, known clinicopathologic parameters of the primary study cohort were included in the univariate Cox regression analysis for OS. Clinicopathologic factors with p < 0.1 in the univariate analysis were further performed the multivariate Cox regression analysis. The results indicated that advanced age, high TNM stage, lymph node metastasis, recurrence, and high-grade TB were independent poor prognostic factors for OS, while TLS was an independent favorable prognostic factor for OS. As shown in **Table 3**, univariate and multivariate Cox regression analyses were also performed for DFS. The results demonstrated that advanced age, high TNM stage, and high-grade TB were independent poor prognostic factors for DFS, whereas TLS was an independent favorable prognostic factor for DFS.

**Table 2:** Cox regression model for OS of OSCC patients in primary cohort.

Clinicopathologic features	Univariate analysis		Multivariate analysis	
	HR (95% CI)	*p*	HR (95% CI)	*p*
Age, years				
< 60	1		1	
≥ 60	2.01 (1.29 ~ 3.15)	0.002*	1.62 (1.03 ~ 2.54)	0.038*
Gender				
Female	1			
Male	1.25 (0.78 ~ 2.02)	0.356		
DOI				
≤ 5mm	1			
> 5mm	2.17 (1.37 ~ 3.45)	<0.001*	1.01 (0.57 ~ 1.79)	0.961
TNM stage				
I/II stage	1			
III/IV stage	5.16 (3.28 ~ 8.10)	<0.001*	2.26 (1.11 ~ 4.61)	0.024*
Pathologic differentiation				
Well	1			
Moderately/Poorly	1.09 (0.70 ~ 1.71)	0.698		
Lymph node metastasis				
No	1			
Yes	6.71 (4.21 ~ 10.69)	<0.001*	2.89 (1.39 ~ 6.03)	0.005*
Relapse				
No	1			
Yes	3.56 (2.27 ~ 5.59)	<0.001*	2.69 (1.68 ~ 4.30)	<0.001*
TB grade				
low grade	1		1	
high grade	3.56 (1.99 ~ 6.35)	<0.001*	2.68 (1.48 ~ 4.86)	0.001*
TLS maturity				
None/E-TLS	1		1	
PFL-TLS/SFL-TLS	0.43 (0.23 ~ 0.80)	0.008*	0.37 (0.20 ~ 0.69)	0.002*

*represents p<0.05.

OS, overall survival; OSCC, oral squamous cell carcinoma; CI, confidence interval; HR, hazard ratio; DOI, depth of invasion; TNM, tumor-lymph node-metastasis; TB, tumor budding; TLS, tertiary lymphoid structures; E-TLS, early tertiary lymphoid structure; PFL-TLS, primary follicle like tertiary lymphoid structure; SFL-TLS, secondly follicle-like tertiary lymphoid structure.

**Table 3:** Cox regression model for DFS of OSCC patients in primary cohort.

Clinicopathologic features	Univariate analysis		Multivariate analysis	
	HR (95% CI)	*p*	HR (95% CI)	*p*
Age, years				
< 60	1		1	
≥ 60	1.74 (1.20 ~ 2.50)	0.003*	1.60 (1.11 ~ 2.31)	0.013*
Gender				
Female	1			
Male	0.89 (0.62 ~ 1.30)	0.554		
DOI				
≤ 5mm	1			
> 5mm	1.67 (1.16 ~ 2.41)	0.006*	1.26 (0.82 ~ 1.92)	0.292
TNM stage				
I/II stage	1			
III/IV stage	2.74 (1.89 ~ 3.97)	<0.001*	2.39 (1.63 ~ 3.51)	<0.001*
Pathologic differentiation				
Well	1			
Moderately/Poorly	0.93 (0.65 ~ 1.34)	0.71		
TB grade				
low grade	1		1	
high grade	2.41 (1.58 ~ 3.67)	<0.001*	2.02 (1.31 ~ 3.12)	0.001*
TLS maturity				
None/E-TLS	1		1	
PFL-TLS/SFL-TLS	0.68 (0.44 ~ 1.06)	0.087	0.61 (0.39 ~ 0.94)	0.025*

*represents p<0.05.

DFS, disease-free survival; OSCC, oral squamous cell carcinoma; CI, confidence interval; HR, hazard ratio; DOI, depth of invasion; TNM, tumor-lymph node-metastasis; TB, tumor budding; TLS, tertiary lymphoid structures; E-TLS, early tertiary lymphoid structure; PFL-TLS, primary follicle like tertiary lymphoid structure; SFL-TLS, secondly follicle-like tertiary lymphoid structure.

### Correlation of the TB/TLS index with clinical outcomes in OSCC

Different scores were assigned to TB and TLS in cases from Study Cohort A. According to the ITBCC classification criteria, Bd1 was assigned 1 point, Bd2–2 points, and Bd3–3 points. Based on TLS maturation levels, the absence of TLS and E-TLS were assigned 1 point, PFL-TLS 2 points, and SFL-TLS 3 points.

The optimx() function was used to compare the discriminative ability of different TB-TLS parameter forms for survival outcomes. Six candidate TB-TLS composite forms were evaluated in the training set (**Supplementary Table 1**). The TB/TLS quotient form was selected based on both statistical performance (highest C-index and log-rank χ²) and biological rationale. The optimal cutoff was determined to be 1 using 10-fold cross-validation in the training set. To validate robustness and eliminate circularity, the TB/TLS index and cutoff = 1 were fixed in the training set and tested in the held−out 30% test set of Cohort A. The index remained significantly prognostic (log-rank χ² = 6.8, *p* = 0.0089; C-index = 0.686), confirming its generalizability and absence of overfitting.

**Figure 4:**
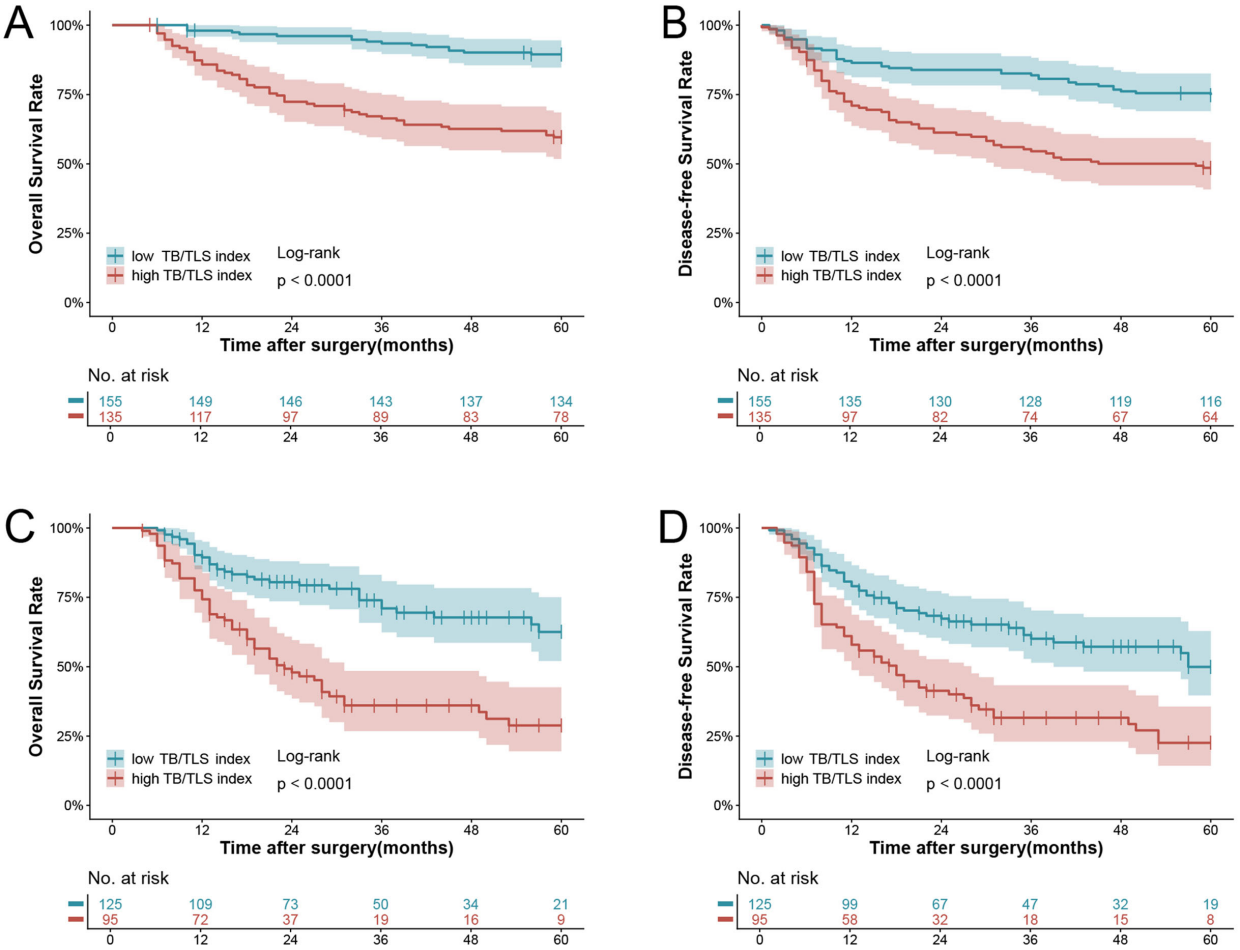
Kaplan-Meier curves for OS and DFS of OSCC patients. **(A, B)** OS and DFS curves stratified by the TB/TLS index in OSCC patients from cohort A; **(C, D)** OS and DFS curves stratified by the TB/TLS index in OSCC patients from cohort **(B)** The colored areas in the figures represent the 95% confidence intervals. OS, overall survival; DFS, disease-free survival; OSCC, oral squamous cell carcinoma; TB, tumor budding; TLS, tertiary lymphoid structures.

Patients were stratified into the low TB/TLS index group (≤ 1) and high TB/TLS index group (> 1) based on the TB/TLS index. Subsequent survival analysis was performed according to the TB/TLS index, with survival curves plotted and log-rank tests conducted. In Cohort A, the 5-year OS and DFS rates in the high TB/TLS index group were 48.6% and 31.9%, respectively, which were significantly lower than those in the low TB/TLS index group (89.5% and 74.8%, respectively; p < 0.0001 for both; **Figures 4A, B**). Applying the same grouping criteria to Cohort B, consistent results were observed (**Figures 4C, D**).

Notably, the discriminative ability of the TB/TLS index for survival outcomes was significantly higher than that of TLS alone. To further evaluate the difference in the discriminative ability for survival outcomes between the TB/TLS index and TB alone, log-rank tests were performed on each survival curve, and chi-square values were extracted. The chi-square values of the log-rank tests for OS and DFS survival curves grouped by the TB/TLS index were 36.9 and 22.3, respectively, which were higher than those grouped by TB alone (20.1 and 15.7). The higher chi−square values and better separation of survival curves confirm that the combined index provides more robust risk stratification than either marker alone.

### Construction and validation of nomogram prediction model

**Figure 5:**
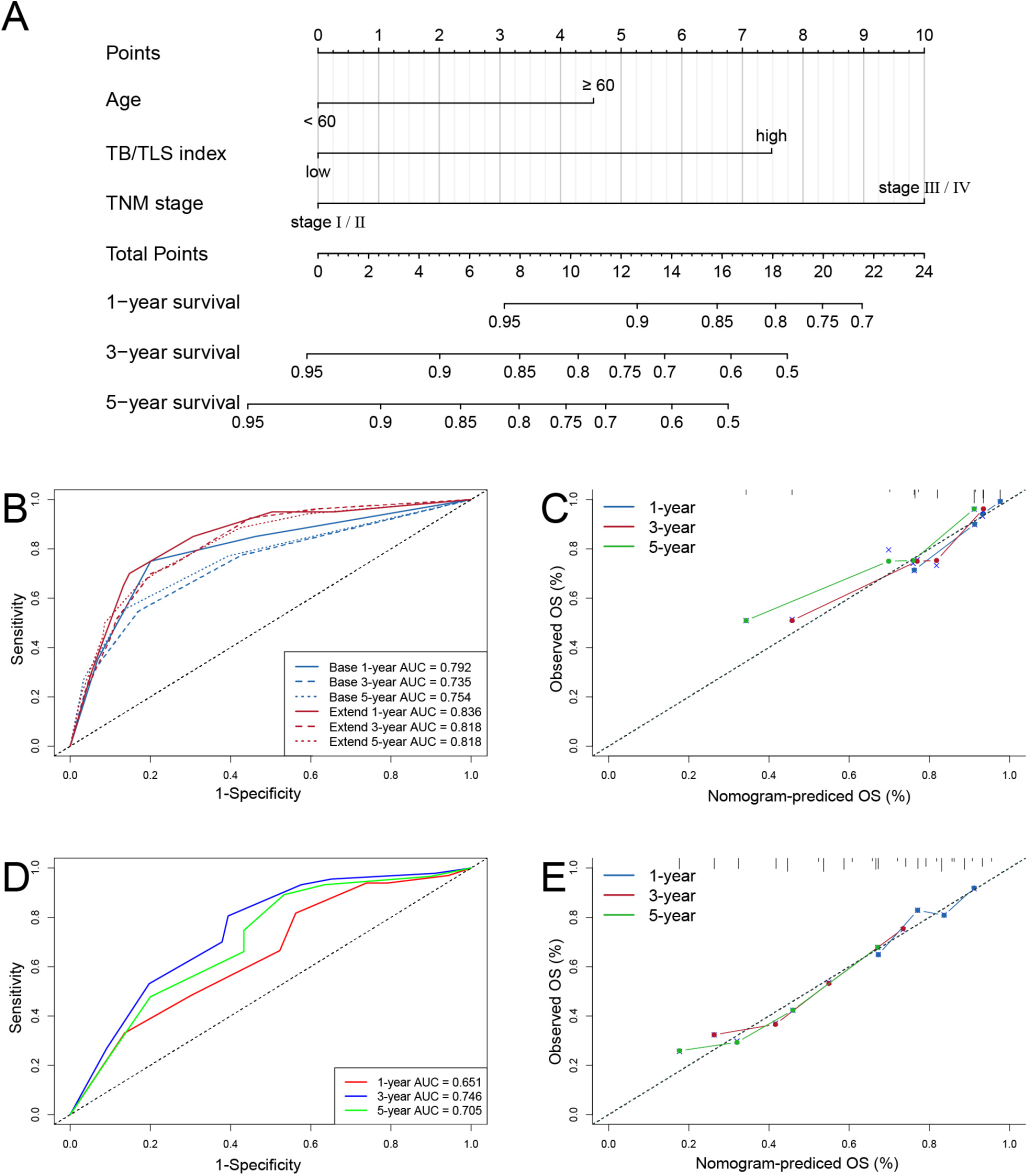
Nomogram prediction model and validation. **(A)** Nomogram for OS in the training cohort; **(B)** ROC curves of the extend predictive model and the base model for OS in the training cohort; **(C)** Calibration curve for OS in the training cohort; **(D)** ROC curve of the predictive model for OS in the validation cohort; **(E)** Calibration curve of the predictive model for OS in the validation cohort. OS, overall survival; TB, tumor budding; TLS, tertiary lymphoid structures; ROC, receiver operating characteristic; AUC, area under the receiver operating characteristic curve.

Based on the results of the multivariate Cox regression analysis in Cohort A, four independent variables were selected: age, TNM stage, TB grade, and TLS maturation level. To maintain alignment with standard clinical staging and avoid redundant information, TNM stage was retained instead of including lymph node metastasis as a separate factor. Lymph node metastasis showed mild multicollinearity with TNM stage (VIF: TNM 2.45, LN 2.36). As the globally recognized gold-standard staging system, TNM stage already comprehensively integrates T, N, and M status; inclusion of both would reduce model parsimony without additional predictive value. Considering that the TB/TLS index had superior discriminative ability for survival outcomes, TB grade and TLS maturation level were integrated into the TB/TLS index to construct a nomogram prediction model (**Figure 5A**). The concordance index (C-index) of the prediction model for OS prediction in the training cohort was 0.773.

To evaluate the impact of the TB/TLS index on the predictive performance of the model, LRT and AIC/BIC difference tests were performed between the extend prediction model incorporating the TB/TLS index and the base model without the TB/TLS index. The results showed that LRT = 15.706 (p < 0.001), and the AIC/BIC values of the extend prediction model were 771.7 and 778.7, respectively, which were lower than those of the base model (785.4 and 790.1). These indicated that the TB/TLS index could significantly improve the predictive ability of the model. Taking 1-year, 3-year, and 5-year OS as the prognostic time points, time-dependent ROC curves were plotted for both the base model and the extend prediction model. The AUC values of the extend prediction model in the training cohort were 0.836, 0.818, and 0.818, respectively, while those of the base model were 0.792, 0.735, and 0.754, suggesting that the TB/TLS index could enhance the model’s predictive ability (**Figure 5B**).

Subsequently, internal validation of the prediction model was performed using 1-year, 3-year, and 5-year OS as the prognostic time points. The line of predicted points on the calibration curve showed good consistency with the ideal curve, indicating that the prediction model had reliable predictive ability (**Figure 5C**). Similarly, the prediction model was used to predict outcomes in the external validation cohort. Time-dependent ROC curves were plotted with 1-year, 3-year, and 5-year OS as the prognostic time points, and the AUC values were 0.651, 0.746, and 0.705, respectively, indicating that the prediction model had moderate predictive ability for this cohort (**Figure 5D**). The line of predicted points on the calibration curve was adjacent to the ideal curve, demonstrating that the prediction model had good predictive ability (**Figure 5E**). Notably, marked baseline differences existed between Cohort A and Cohort B, including higher rates of pT3–4, lymph node metastasis, higher tumor stage, and shorter median follow−up in Cohort B. These differences reflect inherent heterogeneity between a single−institution early−stage cohort and a public database cohort enriched with advanced cases. Therefore, the moderate AUC values in external validation should be interpreted as reflecting both model performance and case−mix heterogeneity.

## Discussion

Prognosis of OSCC varies among different patients. Accurate assessment of survival risk and stratification of patients with distinct clinicopathological factors are the developmental direction of precision medicine. The TME of OSCC contains various pro-tumor and anti-tumor factors, which are closely associated with tumor occurrence, development, metastasis, and anti-tumor immune response (19). In this study, we validated the prognostic value of TB and TLS in OSCC. High-grade TB was associated with poorer OS and DFS, which is consistent with the role of TB as a potential biomarker of tumor invasiveness and metastatic potential (5). TB cells possess EMT and cancer stem cell characteristics, making them more likely to evade immune surveillance, thereby promoting tumor progression and recurrence (8). Conversely, high-maturation TLS was identified as an independent favorable prognostic factor, reflecting the critical role of anti-tumor immunity in OSCC. As a local immune center, TLS facilitates the recruitment, activation, and differentiation of immune cells, enhances the anti-tumor immune response, and suppresses tumor growth. Interestingly, the immunomodulatory effect of TLS is heterogeneous. Immature TLS (E-TLS) may also be associated with cancer progression (20–22).

Compared with analyzing a single component in the TME, researchers have shifted their focus to the dynamic balance between various pro-tumor and anti-tumor factors in the TME, establishing pro-tumor/anti-tumor models that have demonstrated favorable prognostic value and predictive efficacy (23–25). In this study, by integrating the pro-tumor potential of TB and the anti-tumor immune function of TLS, we established a TB/TLS index based on TB grade and TLS maturation level. The TB/TLS index integrates the “offense” of tumor budding and the “defense” of mature TLS, thereby capturing the dynamic invasion–immune balance in the OSCC microenvironment. A high TB/TLS index (indicating that tumor invasiveness is dominant over anti-tumor immunity) was associated with significantly poorer survival outcomes. This index can assess tumor biological behavior more accurately than a single biomarker and enable more precise risk stratification of patients.

In this study, a nomogram was constructed based on independent clinicopathological factors related to prognosis. LRT, AIC/BIC tests, and ROC curves of the prediction model showed that the TB/TLS index improved the prognostic predictive ability of the model. The model exhibited reliable predictive performance in both internal and external data validation, indicating that this prediction model has relatively reliable clinical applicability. The nomogram maintained moderate predictive performance in the external TCGA cohort despite large baseline differences, supporting its potential applicability across heterogeneous OSCC populations. However, given the substantial disparities in stage distribution, lymph node metastasis rates, and follow-up duration between cohorts, the generalizability of our model should be interpreted cautiously. Further prospective validation in well-matched, multi-center cohorts is strongly warranted to confirm real-world clinical utility.

This study also has several limitations. First, the evaluation of TB and TLS in this study was based on H&E staining, which although advantageous in terms of clinicopathological accessibility, has a certain degree of subjectivity. Despite the adoption of standardized assessment, observer bias cannot be completely excluded. Future studies may combine convolutional neural network based deep learning to improve the accuracy and objectivity of TB and TLS evaluation. Second, this is a retrospective study with inherent selection bias. Prospective studies are needed to validate the prognostic value of the TB/TLS index and the nomogram model in a larger and more diverse population. Third, the potential molecular mechanisms underlying the interaction between TB and TLS in OSCC remain unclear. Future basic research could explore the molecular crosstalk between tumor cells in TB and immune cells in TLS, which may provide potential therapeutic targets for OSCC. Finally, the external validation cohort (TCGA) differed significantly from the training cohort in baseline characteristics, including tumor stage, lymph node metastasis, depth of invasion, and follow−up duration. Such heterogeneity may affect the interpretation of external validation performance and limits direct generalization of the model.

Despite these limitations, our study demonstrates the favorable prognostic predictive value of the TB/TLS index in OSCC patients. The nomogram prediction model incorporating the TB/TLS index provides a reliable tool for individualized OS prediction in OSCC patients.

## Conclusion

In this retrospective study of clinical OSCC patient samples, the prognostic value of TB and TLS was validated. The integrated TB/TLS index reflected both tumor aggressiveness and local immune status, and demonstrated superior prognostic discriminatory ability. The nomogram showed moderate predictive performance in the external TCGA cohort despite substantial baseline differences, supporting its potential clinical utility across heterogeneous OSCC populations. These findings may hold clinical significance for risk stratification, treatment optimization, and prognostic assessment in OSCC.

## Data Availability

The raw data supporting the conclusions of this article will be made available by the authors, without undue reservation.
