# Supplementary material for: Predicting survival in oral squamous cell carcinoma via integrated analysis of tumor budding and tertiary lymphoid structures
Source: Front Oncol. 2026 Jun 3;16:1774998. doi: 10.3389/fonc.2026.1774998 (PMC13271958; doi:10.3389/fonc.2026.1774998)
Supplement: Supplementary file 1 [file Table1.docx]

Supplementary Table S1. Performance of six candidate TB-TLS index forms in the training set of Cohort A

| Index | C‑index | Log‑rank χ² | P value |
| --- | --- | --- | --- |
| TB/TLS | 0.697 | 29.3 | <0.0001 |
| log(TB/TLS) | 0.697 | 29.3 | <0.0001 |
| TLS/TB | 0.697 | 6.9 | 0.0087 |
| TB-TLS | 0.696 | 29.3 | <0.0001 |
| TB alone | 0.655 | 4.5 | 0.0346 |
| TLS alone | 0.590 | 7.6 | 0.0059 |

Abbreviation: TB, tumor budding; TLS, tertiary lymphoid structures
